# Deep Artificial Neural Networks and Neuromorphic Chips for Big Data Analysis: Pharmaceutical and Bioinformatics Applications

**DOI:** 10.3390/ijms17081313

**Published:** 2016-08-11

**Authors:** Lucas Antón Pastur-Romay, Francisco Cedrón, Alejandro Pazos, Ana Belén Porto-Pazos

**Affiliations:** 1 Department of Information and Communications Technologies, University of A Coruña, A Coruña 15071, Spain; pastur90@gmail.com (L.A.P-R.); flanciskinho@gmail.com (F.C.); apazos@udc.es (A.P.); 2 Instituto de Investigación Biomédica de A Coruña (INIBIC), Complexo Hospitalario Universitario de A Coruña (CHUAC), A Coruña 15006, Spain

**Keywords:** artificial neural networks, artificial neuron–astrocyte networks, tripartite synapses, deep learning, neuromorphic chips, big data, drug design, Quantitative Structure–Activity Relationship, genomic medicine, protein structure prediction

## Abstract

Over the past decade, Deep Artificial Neural Networks (DNNs) have become the state-of-the-art algorithms in Machine Learning (ML), speech recognition, computer vision, natural language processing and many other tasks. This was made possible by the advancement in Big Data, Deep Learning (DL) and drastically increased chip processing abilities, especially general-purpose graphical processing units (GPGPUs). All this has created a growing interest in making the most of the potential offered by DNNs in almost every field. An overview of the main architectures of DNNs, and their usefulness in Pharmacology and Bioinformatics are presented in this work. The featured applications are: drug design, virtual screening (VS), Quantitative Structure–Activity Relationship (QSAR) research, protein structure prediction and genomics (and other omics) data mining. The future need of neuromorphic hardware for DNNs is also discussed, and the two most advanced chips are reviewed: IBM TrueNorth and SpiNNaker. In addition, this review points out the importance of considering not only neurons, as DNNs and neuromorphic chips should also include glial cells, given the proven importance of astrocytes, a type of glial cell which contributes to information processing in the brain. The Deep Artificial Neuron–Astrocyte Networks (DANAN) could overcome the difficulties in architecture design, learning process and scalability of the current ML methods.

## 1. Introduction

Machine Learning (ML) is a subfield of Artificial Intelligence which attempts to endow computers with the capacity of learning from data, so that explicit programming is not necessary to perform a task. ML algorithms allow computers to extract information and infer patterns from the record data so computers can learn from previous examples to make good predictions about new ones. ML algorithms have been successfully applied to a variety of computational tasks in many fields. Pharmacology and bioinformatics are “hot topics” for these techniques because of the complexity of the tasks. For example, in bioinformatics, ML methods are applied to predict protein structure and genomics (and other omics) data mining. In the case of pharmacology, these methods are used to discover, design and prioritize bioactive compounds, which can be candidates for new drugs [1]. Moreover, ML can be helpful to analyze clinical studies of these compounds, optimize drug forms, and evaluate drug quality [2,3]. The development of a drug has different phases; in the first step a set of molecular representation, or descriptors, are selected. These descriptors represent the relevant properties of the molecules of interest. The encoded molecules are compared to one another using a metric or scoring scheme. Next, the data set is usually divided into three parts: training set, validation set and test set. The final step involves the use of ML methods to extract features of interest that can help to differentiate active compounds from inactive ones. Quantitative Structure-Activity Relationship (QSAR) is used to find relationships between the structure of a compound and its activity, both biological and physicochemical [4]. There are similar mathematical models that look for other relationships, such as Quantitative Structure-Property Relationship (QSPR), Quantitative Structure–Toxicity Relationship (QSTR) or Quantitative Structure–Pharmacokinetic Relationship (QSPkR) [5].

It is of major importance to select the right descriptors to extract valuable features from the input data. The accuracy of these data, and the statistical tools used, are also relevant in the development process [4]. Over the past decades, the ML techniques used in pharmaceutical and bioinformatics applications were “shallow”, with only a few layers of feature transformations. Some of the most used algorithms are: principle component analysis, k-means clustering, decision trees, Support Vector Machines (SVMs) and Artificial Neural Networks (ANNs) [1,4].

The ANNs have been applied to pharmacology and bioinformatics for more than two decades. Historically, the first report on the application of ANNs in these fields was published by Qian and Sejnowski in 1988 [6]. They used ANNs for the prediction of the protein secondary structure. In 1990, Aoyama et al. presented the first report on the application of ANNs to QSAR [7], whereas in 1993, Wikel and Dow disclosed an application of the ANNs in the description of the pruning step of QSAR [8]. An example of an effective application of ANNs was with a data set of HIV-1 reverse transcriptase inhibitors, in the descriptor selection process [9]. Kovalishyn et al. developed a pruning method based on an ANN trained with the Cascade-Correlation learning method in 1998 [10]. These are some examples of early applications of ANNs, but a huge advance had been made in these ML techniques. To get a historical perspective, and to understand in detail the applications of ANNs, and other ML algorithms, to pharmacology and bioinformatics, the reader is referred to these reviews [1,2,3,4,5,11,12,13,14,15,16,17,18].

Although ANNs were soon identified as useful tools for pharmacology and bioinformatics, SVMs and random forest made great progress, dominating the field until recently. The reasons of the limited application of ANNs were: “scarcity” of data, difficulty to understand the features extracted, and the computational cost entailed by the network training. Over the past decade, DNNs have become the state-of-the-art algorithms of ML in speech recognition, computer vision, natural language processing and many other tasks. This was made possible by the advancement in Big Data, Deep Learning and the exponential increase of the chip processing capabilities, especially GPGPUs.

The Big Data term can be understood by the exponential grow of data, since 90% of the data in the world today has been created in the last two years alone. This data explosion is transforming the way research is conducted, making it necessary to acquire skills in the use of Big Data to solve complex problems related to scientific discovery, biomedical research, education, health, national security, among others. In genomic medicine, this can be illustrated by the fact that the first sequenced human genome cost nearly $3 billion, today it can be done for less than $1000. In cancer research, data produced by researchers can be analyzed to support this research. Multiple protein sequences can be analyzed to determine the evolutionary links and predict molecular structures. In Medicine and Bioinformatics, there are numerous opportunities to make the most of the huge amount of data available. Some of the challenges include developing safer drugs, reducing the costs of clinical trials, as well as exploring new alternatives, such novel antibiotics, to fight against resistant microorganisms; and finally, extracting value information from the vast amount of data generated by the public health.

**Figure 1:**
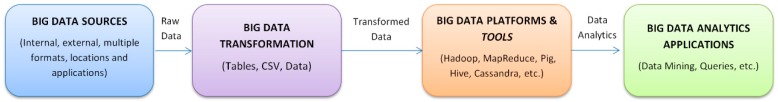
Big Data Workflow.

In order to make the most of the huge amount of information available, different data analysis software frameworks, such as Hadoop, have been created [19]. These frameworks allow the use of simple programming models to process large data sets from thousands of computers. Figure 1 shows a general workflow for Big Data.

DL is a new area of ML research, which is inspired by the brain and data abstraction created by multiple stages of processing. The DL algorithms allow high-level abstraction from the data, and this is helpful for automatic features extraction and for pattern analysis/classification. A key aspect of DL was the development of unsupervised training methods to make the best use of the huge amount of unlabeled data available [11]. Deep Feedforward Neural Networks (DFNN), Deep Belief Networks (DBN), Deep AutoEncoder Networks, Deep Boltzmann Machines (DBM), Deep Convolutional Neural Networks (DCNN) and Deep Recurrent Neural Networks (DRNN) are examples of artificial neural networks with deep learning. They have been applied to fields such as computer vision, automatic speech recognition or natural language processing, where they have been shown to produce state-of-the-art results on multiples tasks, (see Table 1). The idea of building DNNs is not new but there was a historical problem, called “vanishing gradient problem” [20]. It is difficult to train these types of large networks with several layers when the backpropagation algorithm is used to optimize the weights, because the gradients which propagate backwards rapidly diminish in magnitude as the depth of the network increases, thus the weights in the early layers changes very slowly [21]. DNNs have become the leading ML technology for a range of applications since Geoffrey Hinton examined the issues around training large networks [22], and came up with a new approach that had consequences for the cost of training of these networks [23,24]. Over the past decade, a variety of algorithms and techniques have been developed to design and train different architectures of DNN [25,26,27,28,29,30,31].

Finally, GPUs were created to process graphics, especially for gaming and design. Some researchers programmed GPUs using API, but this was a difficult task [33]. In 2007, NVIDIA published “Compute Unified Device Architecture” (CUDA) [34], a programming language based on C to optimize GPGPU application. CUDA allows researchers to make the most of the computing capabilities of GPUs for parallel programming. Nowadays, almost every supercomputer in the TOP500 combines CPUs and GPUs [35]. GPUs are beneficial for DL because the training of DNN is very intensive, so this training can be parallelize with GPUs and a performance improvement greater than 10× can be obtained. However, the ongoing work on design and construction of neuromorphic chips should be pointed out, as they represent a more efficient way to implement DNNs [36]. The neuromorphic chips attempt to mimic the neuronal architectures present in the brain in order to reduce several orders of magnitude in terms of energy consumption and to improve the performance of the information processing. However, to run DNNs in a neuromorphic chip, they should be mapped in a spiking artificial neural network (SNN) [37].

In this review, the main architectures of DNNs and their applications in pharmacology and Bioinformatics are presented. The future need for neuromorphic hardware for DNNs is also discussed, and the two most advanced chips that have already implemented DL are reviewed: IBM TrueNorth and SpiNNaker. In addition, this work points out the importance of considering astrocytes in DNNs and neuromorphic chips, given the proven importance of this type of glial cells in the brain.

## 2. Deep Artificial Neural Networks in Pharmacology and Bioinformatics

DL is a branch of ML that attempts to mimic the information processing in layers of neurons in the neocortex. DNNs are trained to learn to recognize patterns in digital representations of sounds, images, and other data. Usually, there is an unsupervised pre-training process, which helps to initialize the weights. There are different DNN architectures, but in this review, only the most representative types are briefly explained, we divided them in: Deep Auto-Encoder Networks (DAENs), Deep Convolutional Neural Networks (DCNNs) and Deep Recurrent Neural Networks (DRNNs). DAENs encompass Deep Feedforward Neural Networks (DFNNs), Deep Belief Networks (DBNs), Deep Restricted Boltzmann Machines (DRBMs) and Deep Auto-Encoder Networks. There are differences between these architectures, but they have in common big differences with respect to DCNNs and DRNNs. These differences are highlighted, and some featured applications in Pharmacology and Bioinformatics of each architecture are presented in Table 2. For a more detailed analysis of the DL architecture, the differences, the training and the historical perspective, the reader should refer to these reviews [25,26,27,28,29,30,31].

### 2.1. Deep Auto-Encoder Networks

As previously mentioned, the breakthrough of how to train DAENs was made by Hinton and his team [23,24]. DAENs are models composed of multiple layers of neurons, trained one by one, and could be stacked to as many as possible layers. Different DL architectures, such as DFNN, DBN, DRBM and Deep Auto-Encoder Networks, were grouped together by us. There are some differences between these architectures, but in general the idea of DAENs is stacking various layers of neurons, to be pre-trained one by one, using each layer to train the next one. In the first layer, neurons learn to recognize low level features. In an image, they could recognize basic forms such as lines, edges, etc. The intermediate layers detect more abstract features, the ones detected depending on the data set used to train the networks. For example, if a data set of faces is used, the intermediate layers can recognize parts of the faces like eyes, mouth or ears. Finally, the last layer is trained to detect the most abstract features, for example to recognize a person, a car or an animal in an image.

Usually, the training falls into two steps: the first step is layer-wise pre-training and the second step is fine-tuning. Compared to how a neural network is traditionally trained, the first step can also be seen as a clever way of initialization, whereas the second step can be as simple as backpropagation, depending on the model to be trained.

#### 2.1.1. Pharmacology

A team led by George Dahl, from Hinton’s group, won the Merck Molecular Activity Challenge organized by Kaggle in 2012, indicating the high potential of DL in drug design, and drawing the attention of the pharmacology community. Merck’s data sets include on-target and ADME (absorption, distribution, metabolism, and excretion) activities. Each molecule is represented by a list of features, i.e., descriptors in QSAR nomenclature The DAEN have three hidden layers, each layer having 2000 neurons, so the network has over 24 million tunable values. Generative unsupervised pretraining and the procedure of dropout are used to avoid overfitting [38,39].

However, the small scale of Merck’s data set, 11,000 descriptors, 164,000 compounds, and 15 drug targets, does not allow assessing the value of DL in drug target prediction. In 2014, Unterthiner et al. analyzed the performance in a bigger data set, similar to the in-house data of pharmaceutical companies [40]. The ChEMBL database has 13 million compound descriptors, 1.3 million compounds, and 5000 drug targets. DAEN was compared to seven target prediction methods, including two commercial predictors, three predictors deployed by pharmaceutical companies, and ML methods that could scale to this data set. DAEN outperformed all the other methods and surpassed the threshold to make VS possible. This showed the potential of DL to become a standard tool in industrial drug design [40]. Unterthiner’s team won Tox21 Data Challenge within the “Toxicology in the 21st Century” (Tox21) initiative launched by the United States agencies (NIH, EPA and FDA). The goal of this challenge was to assess the performance of computational methods in predicting the toxicity of chemical compounds. The DAEN used by Unterthiner’s team clearly outperformed all the other participating methods [41]. In the first column of Table 3 this method is shown, and the area under the Receiver Operating Characteristic curve (AUC) value is presented in the second column. The last column shows the *p*-value of a paired Wilcoxon with the alternative hypothesis that the DAEN has on average a larger AUC [40].

Dahl et al. also performed an experiment on assay results deposited in PubChem (see Table 4); they used a DAEN to learn a function that predicts activities of compounds for multiple assays at the same time, which is called multi-task. Cellular and biochemical assays were included in the dataset. Multiple related assays, for example assays for different families of cytochrome P450, were used [42,43].

In a series of empirical studies performed by Google and Stanford, several aspects of the use of massively multi-task framework for VS were analyzed. To characterize each molecule, Extended Connectivity Fingerprints (ECFP4) was used. This method decomposes each molecule in fragments that are centered on a non-hydrogen atom. The fragments are labeled with an identifier, and all the identifiers from a molecule are stored into vector of fixed length which represents the molecular fingerprint. The results showed that both additional data and additional tasks improve accuracy. Overall, 259 data sets, containing 37,800,000 experimental data points for 1,600,000 compounds, were used [44].

#### 2.1.2. Bioinformatics

**Figure 2:**
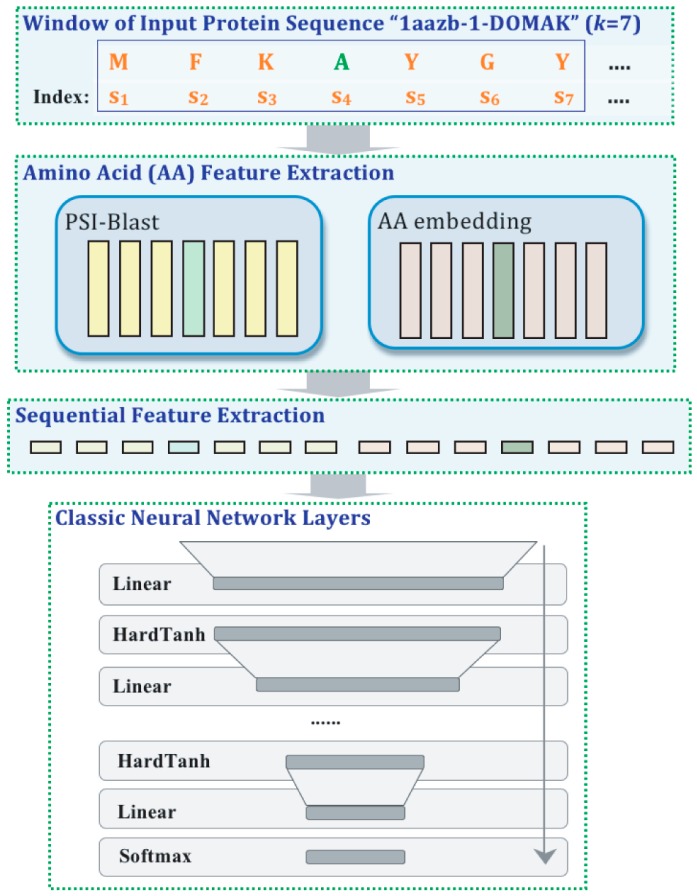
Deep neural network architecture from Yanjun Qi et al. [45]. The input to the first layer is the protein sequence represented by the single-letter amino acid code, for example the letter “A” (in green) represents “Alanine”. This method uses a sliding window input {S_1_, S_2_… S_k_}, in this case *k* = 7. The first layer consists a PSI-Blast feature module and an amino acid embedding module, the green boxes represent the feature vector derived from the Alanine in both modules. In the second layer, the feature vectors are concatenated to facilitate identification of local sequence structure. Finally the derived vector is fed into the Deep Artificial Neural Network.

Yanjun Qi et al. [45] created a DAEN to predict local properties of a protein based on its sequence. Some of the properties predicted were the solvent accessible surface area, transmembrane topology, DNA-binding residues, signal peptides and the secondary structure (see Figure 2). The DAEN used the amino acid sequence as an input to predict the class labels. The method has three levels: first is a layer for the feature extraction from the amino acid; the second is a layer for sequential feature extraction; and, third, different layers of ANN. This method obtained state-of-the-art results [45].

DL architectures could be applied to predict the protein contact map. A group from the University of California used a method with three levels of resolution steps. In the first step, coarse contacts and orientations between elements of the secondary structure were predicted using 2D RNN. Subsequently, to align these elements, a method based on energy was used, and the contact probabilities between residues in strands or α-helices were predicted. In the third step, the information over space and time was integrated to refine the predictions. The DL methods only achieve a 30% of accuracy, but this represents an important improvement over other methods [46]. Eickholt and Cheng predicted contacts between protein residues using a DAEN. The method was evaluated with the official Critical Assessment of protein Structure Prediction (CASP) assessors, and with the cluster accuracy and cluster count metrics. The predictor achieved better results predicting long-range contacts than residue-residue contacts. For the top L/10 long-range contacts, the DAEN obtained a 66% of accuracy, using a neighborhood of size 2 [47,48].

In 2014, Lyons et al. published a paper about the use of a DAEN to predict the backbone Cα angles and dihedrals based on the sequences of proteins. The mean absolute error for the predicted angles was between 34 degrees for τ and 9 degrees for θ. The structures constructed of 10 residue fragments based on the prediction, only differ 1.9 Å in average, measured with the root-mean-square distance [49]. A more complete study, published in Nature, showed the potential of DL for the prediction of the protein secondary structure, solvent accessibility and local backbone angles. To evaluate the DL method, a test data set with 1199 proteins was used. The DAEN predicted the secondary structure of the proteins with 82% accuracy, while the predicted and the real solvent surface area had a 76% correlation. The backbone angles had mean absolute errors between 8 and 32 degrees [50]. DAENs can also be applied to assess the quality of the protein models, and obtain better results than the methods based in energy or scoring functions. A DL method was proposed by Nguyen et al., and it was called DL-Pro. The distance between two residues C-α atoms was used to create a representation that is independent of the orientation. A dataset from the CASP competition was used, and the DL-Pro achieve better results than the state-of-the-art methods [51].

Tan et al. applied DAENs to unsupervised feature construction and knowledge extraction to analyze the gene expression data from a breast cancer database. The constructed features extracted valuable information, from both a clinical and molecular perspective. This DAEN learnt to differentiate samples with a tumor, the state of estrogen receptor, and molecular subtypes [52].

DAENs were trained by a group from the University of California, Irvine, to annotate the pathogenicity of genetic variants using training data consisting of 16M observed variants and 49M simulated variants. This model improved considerably the performance of other methods, around 15% [53].

The genes are very important in all biological processes, and nowadays their study has been facilitated due to the DNA microarray technology. The expression of thousands of genes is measured in one go, and this produces a huge amount of data. Gupta et al. proposed a DL architecture to learn the structure in gene expression, with an application to gene clustering [54].

### 2.2. Deep Convolutional Neural Networks

**Figure 3:**
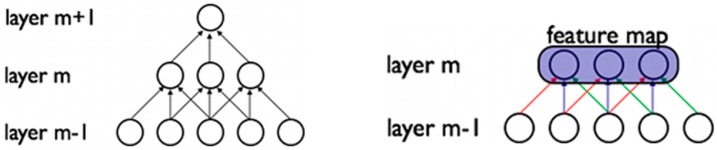
Convolutional layers that extract features of the input to create a feature map. The artificial neurons are represented by the circles, and the weights by the narrows. Weights of the same color are shared, constrained to be identical [56].

The CNN are inspired by the structure of the visual cortex, discovered by Hubel and Wiessel [55], which is formed by a complex pattern of neurons that are sensitive to small sub-regions, creating receptive fields which act as local filters. The natural images, and other types of data, present a strong correlation between nearby pixels, or input data points, and this relationship can be exploited by these receptive fields to extract valuable patterns of features. The CNNs mimic this architecture and have convolutional layers in which each neuron is connected with a subset of neurons of the previous layer [56]. For example, in Figure 3, the neurons of the layer *m* are connected to 3 neurons from the layer *m-*1, therefore each neuron only receives information from the sub-region of the input space.

**Figure 4:**
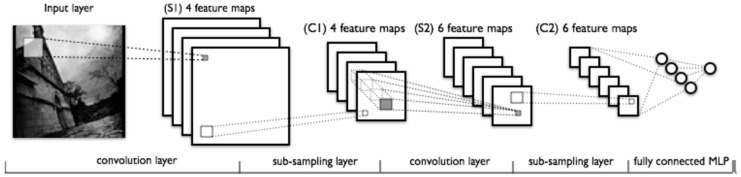
Architecture of a Deep Convolutional Neural Network (DCNN), alternating the convolutional layer and the max-pooling layer (or sub-sampling layer), and finally the fully-connected layer [56].

The CNNs trained with natural images learnt to recognize different patterns in the pixels. Each neuron acts like a filter, but only on a subset of the input space. The neurons from the top layers integrated information from more pixels, thus they can detect more abstract patterns. CNNs [25,26,27,28] were designed to recognize visual patterns from insufficiently preprocessed pixels and can recognize patterns with extreme variability, exhibiting robustness to distortions and transformations. There are three types of layers: convolutional, Max-Pooling and fully-connected (see Figure 4). CNNs are not limited to two-dimension input data, like images, and can be applied to 1, 3 or even more dimensions of data, for example one dimension audio for speech recognition and 3 or 4 dimension for functional magnetic resonance imaging.

#### 2.2.1. Pharmacology

**Figure 5:**
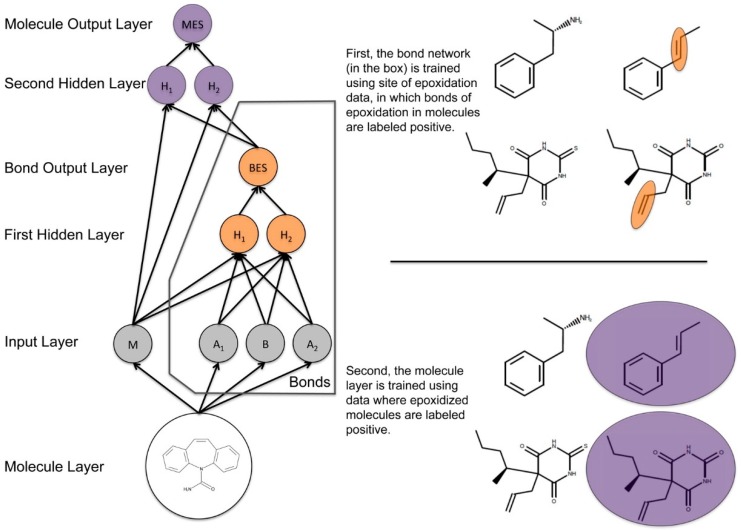
This diagram represents a simplification of the structure of the epoxidation model, which was made up of one input layer, two hidden layers, and two output layers. The actual model had several additional nodes in the input and hidden layers. In the input layer, M represents the molecule input node, B is the bond input node, and two atom input nodes (for each atom associated with the bond). The bond epoxidation score (BES) quantifies the probability that the bond is a site of epoxidation based in the input from the nodes of the first hidden layer (H_1_ and H_2_). The molecule epoxidation score (MES) reflects the probability that the molecule will be epoxidized. This score is calculated with the information from the all molecule-level descriptors and the BES. The bond network and the molecule network are represented in orange and purple respectively [57].

DCNNs have been used to predict drug toxicity both at the atomic and molecular level. Hughes et al. published a study that described a new system, used to predict the formation of reactive epoxide metabolites. This method needs to be combined with additional tools in order to predict the toxicity of drugs. For example, while this model predicts the formation of epoxides, it does not score the reactivity of these epoxides (see Figure 5) [57].

**Figure 6:**
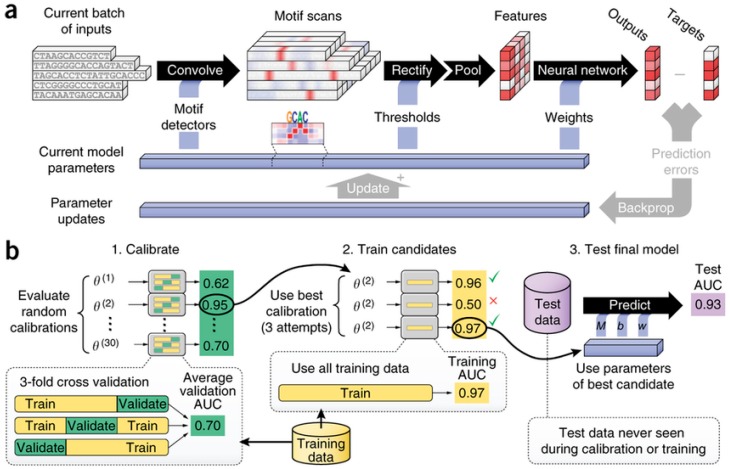
Details of inner workings of DeepBind developed by Alipanahi et al. and its training procedure. In “**a**”, five independent sequences of DNA are being processed in parallel, each composed by a string of letters (C, G, A and T) which represent the nucleotides. The scores are represented in white and red tones, and the outputs are compared to the targets to improve the model using backpropagation; In “**b**”, The Calibration, training, and tasting procedure is represented in more detail [59].

Figure 6 shows how information flowed through the model, which was composed of one input layer, two hidden layers, and two output layers. This model computed a molecule-level prediction for each test molecule as well as predictions for each bond within that test molecule [57].

#### 2.2.2. Bioinformatics

DCNNs were used to predict the target of microRNA, which regulates genes associated with various diseases. Cheng et al. presented a DCNN that outperforms the existing target prediction algorithms and achieves significantly higher sensitivity, specificity and accuracy with values of 88.43%, 96.44% and 89.98%, respectively [58]. DCNNs can also be applied to predict the sequence specificities of DNA and RNA binding proteins. Alipanahi et al. developed a DL approach called DeepBind that outperforms other state-of-the-art methods, even when training on in vitro data and testing on in vivo data (see Figure 6) [59,60].

### 2.3. Deep Recurrent Neural Networks

**Figure 7:**
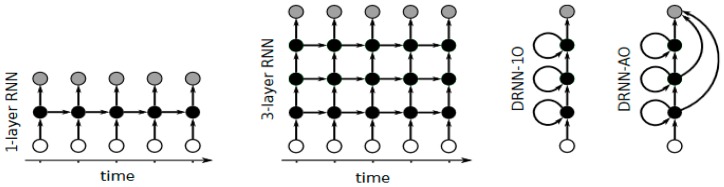
Different Recurrent Neural Networks architectures, the white circles represent the input layers, the black circles the hidden layers, and the grey circles the output layers [65].

RNNs are a type of ANN that has recurrent connections, thus the network represents a directed cycle [61]. The RNNs can exhibit dynamic temporal behavior so they can process sequence of inputs due to their internal memory containing the recurrent connections. This makes them well suited to be applied to tasks like handwriting recognition with unsegmented characters [62] or speech recognition [63]. In a feedforward neural network, the depth is measured as the number of layers between the input and output. Unfortunately, this definition does not apply trivially to a recurrent neural network (RNN) because of its temporal structure. A DRNN is a DNN with recurrent connections in each layer [64,65]. When the network is updated, the information flows in both directions, up and down, thus the sequential information can be learned (see Figure 7). The sequence of updates allows the networks to integrate the information in different time scales, creating a temporal hierarchy.

#### 2.3.1. Pharmacology

Lusci et al. presented a brief overview of some applications of DRNNs aimed at the prediction of molecular properties, such as aqueous solubility. Undirected cyclic graphs are usually used to describe the molecules; however, the RNN typically uses directed acyclic graphs. Therefore, there was a need to develop methods that would address the discrepancy by considering a set of DRNN associated with all possible vertex-centered acyclic orientations of the molecular graph. The results obtained proved that the DRNN performance is equal to or better than the other methods [66].

**Figure 8:**
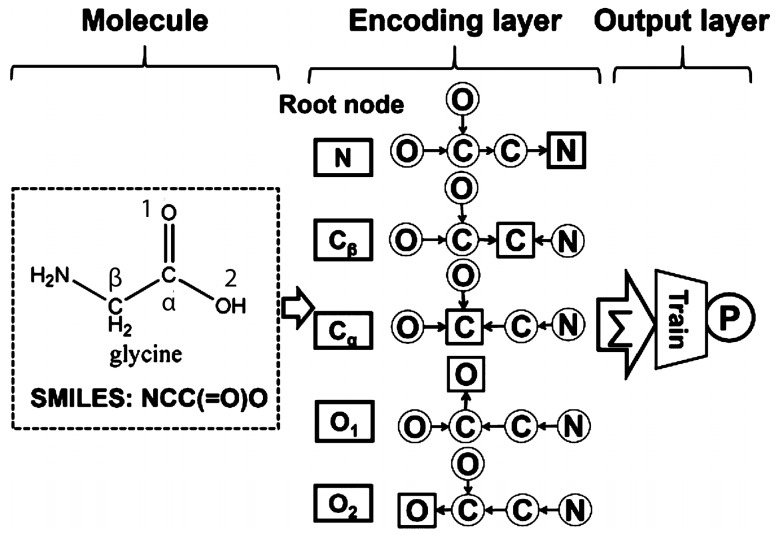
Schematic diagram of Youjun Xu et al. network encoding glycine, first using primary canonical SMILES strucuture. Then, each of the atoms in the SMILES structure is sequentially defined as a root node. Finally, information for all other atoms is transferred along the shortest possible paths, in which case is obtained following the narrows [67].

Over the past 50 years, drug-induced liver injury has cost a huge amount of money to the pharmaceutical companies due to the drug withdrawal caused by this problem. DL methods has been successfully applied to predict drug-induced liver injury Xu et al. compared different DL architectures to predict drug-induced liver injury using four large data sets, and the best results were obtained by a novel type of DRNN (see Figure 8). The structure of glycine is converted into a primary canonical SMILES structure. Subsequently, each of the atoms in the SMILES structure is sequentially defined as a root node. Finally, the information for all the other atoms is transferred along the shortest possible paths. The best model achieved an accuracy of 86.9%, sensitivity of 82.5%, specificity of 92.9%, and area under the curve (AUC) of 0.955 [67].

#### 2.3.2. Bioinformatics

DRNNs can be used to analyze biological sequence data, like predicting the subcellular location of proteins. Sønderby et al. created a DRNN using only the protein sequence, and achieved 92% of accuracy in the prediction of the location of proteins, outperforming the current state-of-the-art algorithms. The performance was improved by the introduction of convolutional filters and the authors experimented with an attention mechanism that let the network focus on specific parts of the protein [68].

## 3. Neuromorphic Chips

Since Alan Turing created the first computer, the progress in computer science has been remarkable. This progress was predicted by Gordon Moore in 1965, who foretold that the number of transistors that could be manufactured on a single silicon chip would double every 18 months to two years. It is known as Moore’s Law, and over the past century it has been accomplished by making transistors increasingly smaller. As CMOS transistors get smaller they become cheaper to make, faster, and more energy-efficient. This win-win scenario has driven the society to a digital era in which computers play a key role in almost every walk and aspect of our lives [22].

However, Moore’s Law has limitations when it comes to shrinking transistors; there is a physical limit in the size of the atom. At this scale, around 1 nm, the properties of the semi-conductor material in the active region of a transistor are compromised by quantum effects like quantum tunneling. In addition, there are also other limitations, such as the energy wall [69,70] and memory wall [71], which denote the high power density and low memory bandwidth [72,73]. There are also economic limitations, since the cost of designing a chip and the cost of building a fabrication facility are growing alarmingly [74].

Trying to avoid some of these limitations, in the early years of this century, all of the major microprocessor manufacturers moved from ever-faster clock speeds to multicore processors. Over the past decade, instead of creating faster single-processor machines, new systems include more processors per chip. Now we have CPUs with multicores, and GPUs with thousands of cores [22].

**Figure 9:**
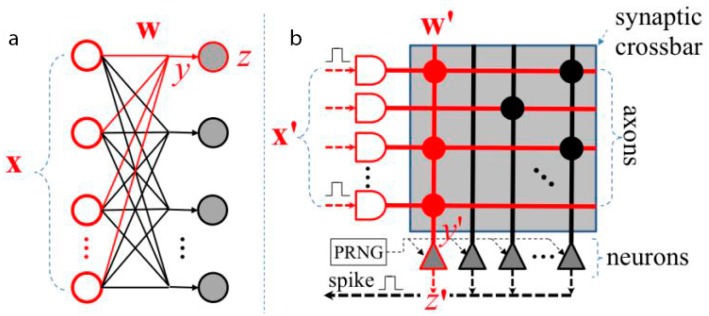
Mapping a Deep Artificial Neural Network (DANN) (**a**) to a neuromorphic chip like the TrueNorth (**b**). The input neurons are represented with the red and white shapes (x and x’), and the output neurons with the grey shapes (z and z’). The weights (w) to the neuron z are approximated using a Pseudo Random Number Generator (PRNG), resulting in the weights (w’) to the neuron z’ in the neuromorphic chip [74].

As already stated, DNNs have become the state-of-the-art algorithms of ML in many tasks. However, both training and execution of large-scale DNNs require vast computing resources, leading to high power requirements and communication overheads. The ongoing work on design and construction of neuromorphic chips, the spike-based hardware platforms resulting from the book about VLSI (Very Large Scale Integration) written by Lynn Conway and Carver Mead, and published in the 1980s [75], offered an alternative by running DNNs with significantly lower power consumption. However, the neuromorphic chips have to overcome hardware limitations in terms of noise and limited weight precision, as well as noise inherent in the sensor signal [36]. Moreover, it is necessary to design the structure, neurons, network input, and weights of DNN during training, to efficiently map those networks to SNN in the neuromorphic chips (see Figure 9) [76].

Focusing on projects involving neuromorphic hardware, the IBM TrueNorth chip [77] is one of the most impressive silicon implementation of DNNs. SpiNNaker, a project developed by the University of Manchester, also achieved excellent results implementing DNNs. Both [78] chips are digital, they compute the information using the binary system. However, some neuromorphic chips are analog, they consist of neuromorphic hardware elements where information is processed with analog signals; that is, they do not operate with binary values, as information is processed with continuous values [22]. In analog chips, there is no separation between hardware and software, because the hardware configuration is in charge of performing all the computation and can modify itself [79]. A good example is the HiCANN chip, developed at the University of Heidelberg, which uses wafer-scale above-threshold analog circuits [80]. There are also hybrid neuromorphic chips, like the Neurogrid from Stanford [81], which seek to make the most of each type of computing. It usually processes in analog and communicates in digital. This review will focus only on digital neuromorphic chips, the IBM TrueNorth and the SpiNNaker chip, because are the most advanced projects, obtained the best results implementing DNNs and published the highest number of technical papers. For further details about other projects and the differences between digital, analog and hybrid neuromorphic chips, the reader should refer to other reviews [82,83].

### 3.1. TrueNorth International Business Machines (IBM)

**Figure 10:**
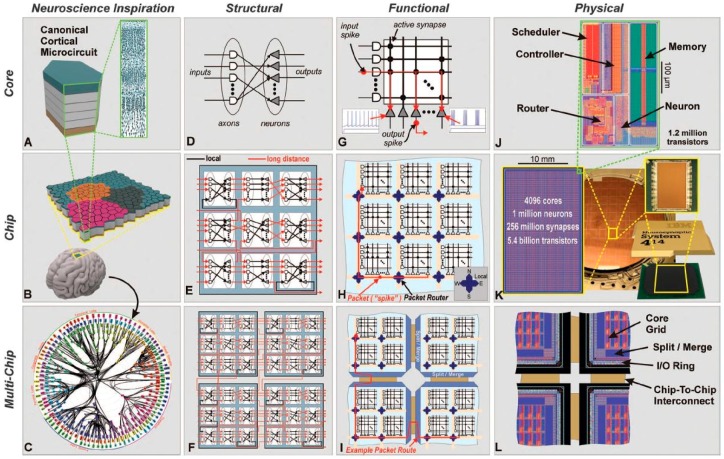
(**A**) The neurosynaptic core is loosely inspired by the idea of a canonical cortical microcircuit; (**B**) A network of neurosynaptic cores is inspired by the cortex’s two-dimensional sheet, the brain regions are represented in different colors; (**C**) The multichip network is inspired by the long-range connections between cortical regions shown from the macaque brain; (**D**–**F**) Structural scheme of the core, chip and multi-chip level. The white shapes represent axons (inputs) and the grey shapes the neurons (outputs); (**G**–**I**) Functional view at different level; (**J**–**L**) Image of the physical layout [77].

The DARPA SyNAPSE (System of Neuromorphic Adaptive Plastic Scalable Electronics) initiative selected and funded the proposal “Cognitive Computing via Synaptronics and Supercomputing (C2S2)” of the Cognitive Computing Group at IBM Research-Almaden directed by Dharmendra Modha [77]. The project is based on the design and creation of a neuromorphic chip called TrueNorth, which has a non-von Neumann architecture. It is characterized by modularity, parallelism and scalability. It is inspired by the brain and its function, low power, and compact volume (see Figure 10). This chip can be used to integrate spatio-temporal and real-time cognitive algorithms for different applications [84]. Currently in the final phase of the project, the researchers created a board with 16 TrueNorth neuromorphic chips, capable of simulating 16 million neurons and four billion synapses. In 2015, they assembled a system consisting of 128 chips and 128 million neurons [85]. The next goal is to integrate 4096 chips into a single rack, which would represent four billion neurons and one trillion synapses, consuming around 4 kW of power [86].

The TrueNorth prototype was created in 2011 [87], and it was a neurosynaptic core with 256 digital leaky integrate-and-fire neurons [37] and up to 256,000 synapses. The core is composed of memory and processor, and the communication takes places through all-or-none spike events. This allows an efficient implementation of a parallel asynchronous communication and Address Event Representation (AER) [88,89]. In this communication system, the neurons have a unique identifier, called address, and when a neuron spikes, the address is sent to other neurons. In 2012, Compass [90] was developed, a simulator to design neural networks to be implemented in the neuromorphic chip. Compass is a multi-threaded, massively parallel functional simulator and a parallel compiler. It uses the C++ language, sends spike events via MPI communication and uses OpenMP for thread-level parallelism. A simulator for GPGPU [91] was also developed. Modha’s team simulated in 2007 the brain of a rat in an IBM BlueGene/L supercomputer [92]. In 2010, they simulated a monkey’s brain [93] in IBM BlueGene/P supercomputers from a network map of long-distance neural connections in the brain obtained with 410 anatomical studies (Collation of Connectivity data on the Macaque brain). Later that same year, they published the results of a simulation with Compass of 2048 billion neurosynaptic cores and 5.4 × 10^11^ neurons and 1.37 × 10^14^ synapses [94]. The execution was 1542× times slower than real time, and 1.5 million Blue Gene/Q supercomputers were needed.

A program in the TrueNorth chips consists of a definition of the inputs and outputs to the network and the topology of the network of neurosynaptic cores. The parameters of the neurons and the synaptic weights should be specified, as well as the inter- and intra-core connectivity [84,95].

The programming paradigm has four levels: The lowest level is the corelet, which represents an abstraction of a TrueNorth program like a blackbox, only showing the inputs and outputs, and hiding the other details. The next level is the Corelet Language which allows the creation and combination of corelets. The validated corelets are included in the Corelet Library and can be reused to create new corelets. This is like a repository and makes up the third level. The last level is the Corelet Laboratory, a programming environment to develop new applications. It is integrated with Compass, the TrueNorth simulator [84].

**Figure 11:**
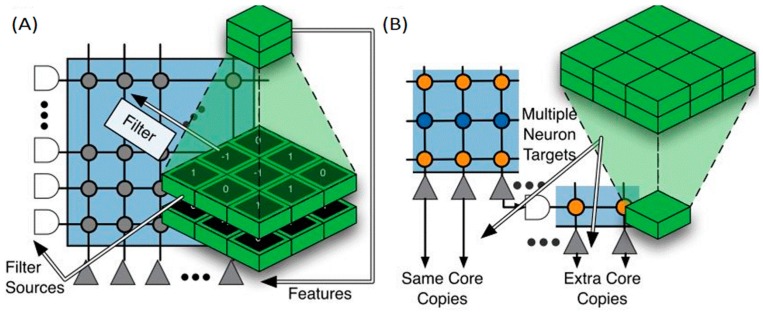
Mapping of a CNN to TrueNorth. (**A**) Convolutional network features for one group at one topographic location are implemented using neurons on the same TrueNorth core, with their corresponding filter support region implemented using the core’s input lines, and filter weights implemented using the core’s synaptic array. The inputs are represented with white shapes, and the grey triangles represent the neurons. The filter used in each case is implemented mapping the matrix of weights (the numbers in the green boxes) into the synaptic array (grey circles); (**B**) For a neuron (blue points) to target multiple core inputs, its output (orange points) must be replicated by neuron copies, recruited from other neurons on the same core, or on extra cores if needed [76].

The corelet library has a collection of several functions that were implemented in the TrueNorth chip verified and parameterized. Some examples are algebraic, logical and temporal functions, convolutions, discrete Fourier transformations and many others. Using these functions different algorithms were implemented in the TrueNorth chip, like CNN (see Figure 11) and Restricted Bolztmann Machines for feature extraction, hidden Markov models, spectral content estimators, liquid state machines, looming detectors, logistic regression, backpropagation and some others. The corelet algorithm can be re-used in different applications, and there are different corelet implementations for the same algorithm, showing the flexibility of the corelet construction [76,96].

TrueNorth was used in different applications, such as recognition of voices, composers, digits, sequences, emotions or eyes. It was also used in collision avoidance and optical flow [96,97].

TrueNorth was also applied to bioinformatics by a group from the University of Pittsburgh, who used the RS130 protein secondary structure data set to predict the local conformation of the polypeptide chain and classified it into three classes: α helices, β-sheets, and coil [74].

### 3.2. SpiNNaker. University of Manchester

SpiNNaker is a project developed at the University of Manchester, whose principal investigator is Steve B. Furber [78]. Within this project, chips, which contain many small CPUs, were produced. Each CPU is designed to simulate about 1000 neurons, such as neural models of leaky integrate and fire or Izhikevich’s model [37], which communicate spike events to other CPUs through a network package. Each chip consists of 18 ARM968 processors, one of them acting as a processor monitor. In 2015, a cabinet with 5760 chips was created, which can simulate 100 million point neurons with approximately 1000 synapses per neuron [98]. The chips are connected with adjacent chips by a two-dimensional toroidal mesh network and each chip has six network ports [99,100,101]. This system is expected to mimic the features of biological neural networks in various ways: (1) native parallelism—each neuron is a primitive computational element within a massively parallel system [102]; (2) spiking communications—the system uses AER, thus the information flow in a network is represented as a time series of neural identifiers [103]; (3) event-driven behavior—to reduce power consumption, the hardware was put into “sleep” mode, waiting for an event; (4) distributed memory—this system uses memory local to each of the cores and an SDRAM local to each chip; and (5) reconfigurability—the SpiNNaker architecture allows on-the-fly reconfiguration [104].

In order to configure a large number of cores, with millions of neurons and synapses, PACMAN [105] was developed. It is a software tool that helps the user to create models, translate and run in SpiNNaker. This allows the user to work with neural languages like PyNN [106] or Nengo [107,108].

The SpiNNaker was created simulate real-time models, but the algorithms had to be defined in the design process, therefore the models were static. In 2013, a paper [109] was published, in which a novel learning rule was presented, describing its implementation into the SpiNNaker system, which allows the use of the Neural Engineering Framework to establish a supervised framework to learn both linear and non-linear functions. The learning rule belongs to the Prescribed Error Sensitivity class.

SpiNNaker supports two types of Deep Neural Networks:

- Deep Belief Networks: These networks of deep learning may be implemented, obtaining an accuracy rate of 95% in the classification of the MNIST database of handwritten digits. Results of 0.06% less accuracy than with the software implementation are obtained, whereas the consumption is only 0.3 W [36,110].
- Convolutional Neural Networks: This type of networks has the characteristic of sharing the same value of weights for many neuron-to-neuron connections, which reduces the amount of memory required to store the synaptic weights. A five-layer deep learning network is implemented to recognize symbols which are obtained through a Dynamic Vision Sensor. Each ARM core can accommodate 2048 neurons. The full chip could contain up to 32,000 neurons. A particular ConvNet architecture was implemented in SpiNNaker for visual object recognition, like poker card symbol classification [111].

Currently, there are no applications in pharmacology or bioinformatics, but SpiNNaker showed its potential by implementing DNNs and DCNNs to visual recognition and robotics. In the future, it could be trained in drug design, protein structure prediction or genomic, and other omics, data mining.

## 4. Discussion

**Figure 12:**
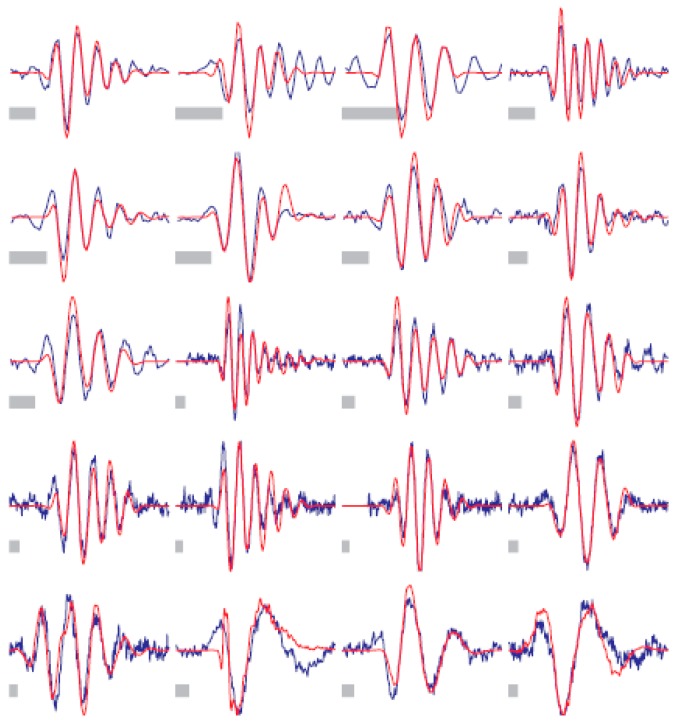
Sparse coding applied to audio. In red 20 basis functions learned from unlabeled audio, in blue the functions from cat auditory nerve fibers [113].

As was pointed out, DNNs have become the state-of-the-art algorithms of ML in speech recognition, computer vision, natural language processing and many other tasks (see Table 1) [26,27]. According to the results obtained, DNNs match the human capabilities, and even surpass them on some tasks. Besides, the inner work of DNNs has similarities with the processing of information in the brain. The pattern of activation of the artificial neurons is very similar to that observed in the brain due to the sparse coding used, which may, for example, be applied to audio to obtain almost exactly the same functions (see Figure 12). In the case of images, it was also shown that the functions learned in each layers were similar to the patterns recognized by each layer of the human visual system (V1 and V2).

This review analyzed applications in pharmacology and bioinformatics (see Table 2). DNNs can be used in the drug discovery, design and validation processes, ADME properties prediction and QSAR models. They also can be applied to the prediction of the structure of proteins and genomic, and other omics, data mining. All these applications are very intensive from a computational perspective, thus DNNs are very helpful because of their ability to deal with Big Data. Besides, DL complement the use of other techniques, for example the quality and success of a QSAR model depend strictly on the accuracy of input data, selection of appropriate descriptors and statistical tools, and most importantly validation of the developed model. Feature extraction from the descriptor patterns is the decisive step in the model development process [4].

Regarding architectures, nowadays, the largest DNN has millions of artificial neurons and around 160 billion parameters [112]. Building large networks will improve the results of DL, but the development of new DL architectures is a very interesting way to enhance the capabilities of the networks. For example, the latest DRNN architectures with “memory” show excellent results in natural language processing, one of the hardest task for ML [26,27,28,29,31].

Some authors, such as Ray Kurzweil [114], claim that the exponential growth based on Moore’s Law and The Law of Accelerating Returns [115] will be maintained, therefore, in the next decades, building a machine with a similar number of neurons as the human brain, of around 86 billion neurons, should be possible. As previously mentioned, there are some physical limitations to the current architecture of computers, such as the memory wall [69,70] and energy wall [71], which denote the high power density and low memory bandwidth [72,73]. There are also economic limitations; the cost of designing a chip and the cost of building a fabrication facility are growing alarmingly [74]. However, these limitations will probably be surpassed using other technologies and architectures, like GPU clusters or networks of Neuromorphic chips. It was historically calculated that the human brain computes approximately 20 billion operations per second [116,117,118,119]. Some authors think that these values underestimate the brain capacity, and calculated around 10^21^ operations per second [120]. However, reaching the human brain capacity is not enough, because one of the main features of the brain is its connectivity of the billions of cells that forms trillions of synapses. Natural evolution has molded the brain for millions of years, creating a highly complex process of development. This was remarkably pointed out by Andrew Ng, neurons in the brain are very complex structures, and after a century of study the researchers still are not able to fully understand how they work. The neurons in the ANN are simple mathematical functions that attempt to mimic the biological neurons. However, the artificial neurons only reach the level of loose inspiration. Consequently, reaching the level of human brain computation will not necessarily mean that the future computers will surpass human intelligence. In our opinion, the advances in understanding the human brain will be more important in order to make a breakthrough that will lead us to new types of DNNs.

**Figure 13:**
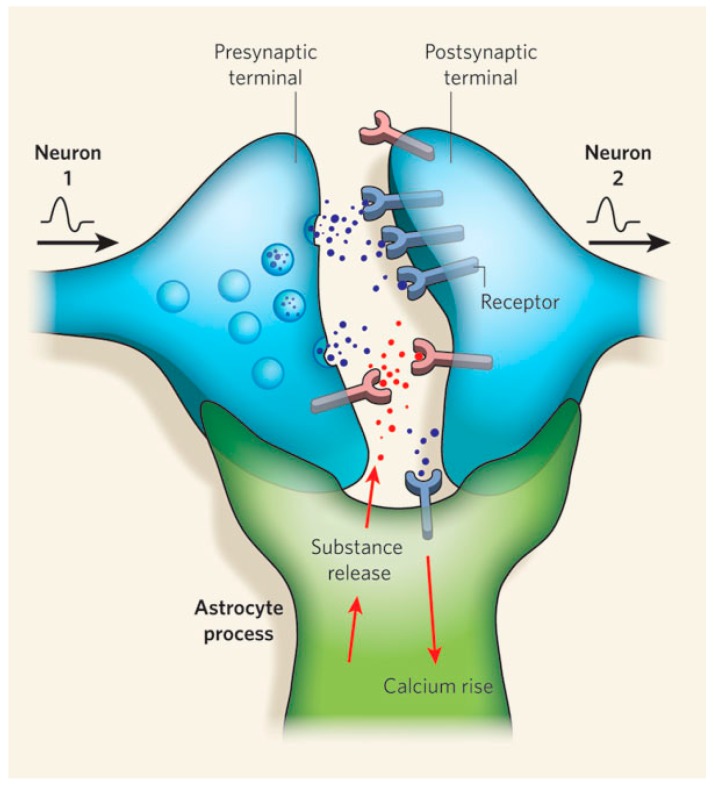
Tripartite synapse represented by a presynaptic neuron, postsynaptic neuron and perisynaptic astrocyte (astrocyte process). The presynaptic neuron release neurotransmitters that are received by the postsynaptic neuron or the perisynaptic astrocyte [129].

In this regard, it should be pointed out that the human brain is composed of neurons, but also glial cells, and there is almost the same number of both [121]. More importantly, over the past decade, it has been proven that astrocytes, a type of glial cells of the central nervous system, actively participate in the information processing in the brain. There are many works published over the past two decades on multiple modes of interaction between neurons and glial cells [122,123,124,125]. Many studies suggest the existence of bidirectional communication between neurons and astrocytes, a type of glial cells of the central nervous system [126,127]. This evidence has led to the proposal of the concept of tripartite synapse [128], formed by three functional elements: presynaptic neuron, postsynaptic neuron and perisynaptic astrocyte (see Figure 13).

The relation between these three elements is very complex and there are different pathways of communication: astrocytes can respond to different neurotransmitters (glutamate, GABA, acetylcholine, ATP or noradrenaline) [130] liberating an intracellular Ca^2+^ signal, known as calcium wave that could be transmitted to other astrocytes through GAP junctions. In addition, astrocytes may release gliotransmitters that activate presynaptic and postsynaptic neuronal receptors, leading to a regulation of the neural excitability, synaptic transmission, plasticity and memory [131,132]. The possibility of a quad-partite synapse, in which microglia are engaged [133], has recently been proposed.

In addition, there is interesting scientific evidence that suggests the important role of glial cells in the intelligence of the species. Although there are no major differences between neurons of different species of mammals, the glial cells have evolved in the evolutionary chain. For example, a rodent’s astrocytes may include between 20,000 and 120,000 synapses, while a human’s may include up to two million synapses [134,135]. Not only should the complexity of the astrocytes be pointed out, but also their size. Human astrocytes have a volume 27 times greater than the same cells in the mouse’s brain [134,135]. Besides, the ratio of glial cells to neurons increased along the evolutionary chain. One of the most striking research events has been the discovery of a single glial cell for every 30 neurons in the leech. This single glial cell receives neuronal sensory input and controls neuronal firing to the body. As we move up the evolutionary ladder, in a widely researched worm, *Caenorhabditis elegans*, glia cells are 16% of the nervous system. The fruit fly’s brain has about 20% glia. In rodents such as mice and rats, glia cells make up 60% of the nervous system. The nervous system of the chimpanzee has 80% glia, while the human 90%. The ratio of glia to neurons increases with our definition of intelligence [123]. The number of astrocytes per neuron also increases as we move up the evolutionary ladder, humans having around 1.5 astrocytes per neuron [136].

Furthermore, the ratio of glial cells to neurons varies in different brain regions. In the cerebellum, for instance, there are almost five times more neurons than astrocytes. However, in the cortex, there are four times more glial cells than neurons [121,137]. All these data suggest that the more complex the task, performed, by either an animal or a brain region, the greater the number of glial cells involved.

Currently, there are two projects aimed at implementing astrocytes in neuromorphic chips, one is BioRC developed by the University of Southern California [138,139,140,141] and the other project is carried out by the University of Tehran and University of Kermanshah, Iran [142,143,144]. Moreover, the RNASA-IMEDIR group from the University of A Coruña developed an Artificial Neuron-Glia Network (ANGN) incorporating two different types of processing elements: artificial neurons and artificial astrocytes. This extends classical ANN by incorporating recent findings and suppositions regarding the way information is processed via neural and astrocytic networks in the most evolved living organisms [145,146,147,148,149]. In our opinion, neurons are specialized in transmission and information processing, whereas glial cells in processing and modulation. Besides, glial cells play a key role in the establishment of synapses and neural architecture. That is why it would be interesting to combine these two types of elements in order to create a Deep Artificial Neuron–Astrocyte Network (DANAN).

## 5. Conclusions

DNNs represent a turning point in the history of Artificial Intelligence, achieving results that match, or even surpass the human capabilities in some tasks. These results motivated major companies like Google, Facebook, Microsoft, Apple and IBM to focus their research on this field. Nowadays, DNNs are used every day unknowingly, since in our smartphones there are numerous applications based on Deep Learning. For example, some cameras use a DNN to perform face recognition, while others employ a voice recognition piece of software, which is also based on DL. There are many other applications with DNNs that perform state-of-the-art results.

Pharmacology and bioinformatics are very interesting fields for DL application, because there is an exponential growth of the data. There is a huge potential in applying DNNs in the process of drug discovery, design and validation that could improve performance and greatly reduce the costs. However, the most promising area is genomics, and other omics, like proteomics, transcriptomics or metabolomics. These types of data are so complex that it is almost impossible for humans to extract valuable insights. Thus, the use of DNNs would be necessary to extract the information useful to understand the relationships between the DNA, epigenetics variations, and different diseases.

Consequently, scientific and economic interests have led to the creation of numerous R&D projects to keep improving DNNs. Developing new hardware architectures is also important in order to improve the current CPUs and GPUs. The neuromorphic chips represent a great opportunity to reduce the energy consumption and enhance the capabilities of DNNs, being very helpful to process a vast volume of information generated by the Internet of Things. Besides, using neuromorphic chips may lead to the creation of a large-scale system that would attempt to represent an Artificial General Intelligence, moving from the current Artificial Narrow Intelligence.

Finally, it would be of great interest to create networks with two types of processing elements, to create DANANs that will work more similarly to the human brain. This should be considered a very resourceful way of improving the current systems, and our group’s objective is to implement this first type of DANAN. This type of networks will consider the proven capabilities of the glial cells in the processing of information, regulation of the neural excitability, synaptic transmission, plasticity and memory, to create more complex systems that could bring us closer to an Artificial General Intelligence.

## Figures and Tables

**Table 1:** Deep Artificial Neural Networks Achievements. Adapted from a slide developed by Yann Lecun, Facebook and NYU [32].

Task (Year)	Competition
Handwriting recognition (2009)	MNIST (many), Arabic HWX (IDSIA)
Volumetric brain image segmentation (2009)	Connectomics (IDSIA, MIT)
OCR in the Wild (2011)	StreetView House Numbers (NYU and others)
Traffic sign recognition (2011)	GTSRB competition (IDSIA, NYU)
Human Action Recognition (2011)	Hollywood II dataset (Stanford)
Breast cancer cell mitosis detection (2011)	MITOS (IDSIA)
Object Recognition (2012)	ImageNet competition (Toronto)
Scene Parsing (2012)	Stanford bgd, SiftFlow, Barcelona datasets (NYU)
Speech Recognition (2012)	Acoustic modeling (IBM and Google)
Asian handwriting recognition (2013)	ICDAR competition (IDSIA)
Pedestrian Detection (2013)	INRIA datasets and others (NYU)
Scene parsing from depth images (2013)	NYU RGB-D dataset (NYU)
Playing Atari games (2013)	2600 Atari games (Google DeepMind Technologies)
Game of Go (2016)	AlphaGo vs. Human World Champion (Google DeepMind Technologies)

**Table 2:** Applications of different Deep Neural Networks (DNNs) architectures.

Network Architecture	Pharmacology	Bioinformatics
DAEN	[1,2,3,4,5,6,7,23]	[8,9,10,11,12,13,14,15,16,17]
DCNN	[18]	[19,20,21]
DRNN	[22,23]	[24]

**Table 3:** Performance of target prediction methods analyzed by Unterthiner et al., in terms of mean AUC (Area Under the Receiver Operating Characteristic curve) across targets [40].

Method	AUC	*p*-Value
Deep Auto-Encoder Network	0.830	–
Support Vector Machine	0.816	1.0 × 10^−7^
Binary Kernel Discrimination	0.803	1.9 × 10^−67^
Logistic Regression	0.796	6.0 × 10^−53^
k-Nearest neighbor	0.775	2.5 × 10^−142^
Pipeline Pilot Bayesian Classifier	0.755	5.4 × 10^−116^
Parzen-Rosenblatt	0.730	1.8 × 10^−153^
Similarity Ensemble Approach	0.699	1.8 × 10^−173^

**Table 4:** List of assays from Pubchem that were used for the study of Dahl et al. [42,43].

Article Identifier	Assay Target/Goal	Assay Type	#Active	#Inactive
1851(2c19)	Cytochrome P450, family 2, subfamily C, polypeptide 19	Biochemical	5913	7532
1851(2d6)	Cytochrome P450, family 2, subfamily D, polypeptide 6, isoform 2	Biochemical	2771	11,139
1851(3a4)	Cytochrome P450, family 3, subfamily A, polypeptide 14	Biochemical	5266	7751
1851(1a2)	Cytochrome P450, family 1, subfamily A, polypeptide 2	Biochemical	6000	7256
1851(2c9)	Cytochrome P450, family 2, subfamily C, polypeptide 9	Biochemical	4119	8782
1915	Group A Streptokinase Expression Inhibition	Cell	2219	1017
2358	Protein phosphatase 1, catalytic subunit, α isoform 3	Biochemical	1006	934
463213	Identify small molecule inhibitors of tim10-1 yeast	Cell	4141	3235
463215	Identify small molecule inhibitors of tim10 yeast	Cell	2941	1695
488912	Identify inhibitors of Sentrin-specific protease 8 (SENP8)	Biochemical	2491	3705
488915	Identify inhibitors of Sentrin-specific protease 6 (SENP6)	Biochemical	3568	2628
488917	Identify inhibitors of Sentrin-specific protease 7 (SENP7)	Biochemical	4283	1913
488918	Identify inhibitors of Sentrin-specific proteases (SENPs) using a Caspase-3 Selectivity assay	Biochemical	3691	2505
492992	Identify inhibitors of the two-pore domain potassium channel (KCNK9)	Cell	2094	2820
504607	Identify inhibitors of Mdm2/MdmX interaction	Cell	4830	1412
624504	Inhibitor hits of the mitochondrial permeability transition pore	Cell	3944	1090
651739	Inhibition of Trypanosoma cruzi	Cell	4051	1324
615744	NIH/3T3 (mouse embryonic fibroblast) toxicity	Cell	3102	2306
652065	Identify molecules that bind r (CAG) RNA repeats	Cell	2966	1287

## Abbreviations

ADME	Absorption, Distribution, Metabolism, and Excretion
AER	Address Event Representation
ANGN	Artificial Neuron-Glia Networks
ANN	Artificial Neural Networks
AUC	Area Under the Receiver Operating Characteristic Curve
CASP	Critical Assessment of protein Structure
CNN	Convolutional Neural Networks
CPU	Central Processing Unit
CUDA	Compute Unified Device Architecture
DAEN	Deep Auto-Encoder Networks
DANAN	Deep Artificial Neuron–Astrocyte Networks
DBN	Deep Belief Networks
DCNN	Deep Convolution Neural Networks
DFNN	Deep Feedforward Neural Networks
DL	Deep Learning
DNN	Deep Artificial Neural Networks
DBM	Deep Boltzmann Machines
DRNN	Deep Recurrent Neural Networks
ECFP4	Extended Connectivity Fingerprints
GPGPUs	General-Purpose Graphical Processing Units
GPU	Graphical Processing Unit
ML	Machine Learning
QSAR	Quantitative Structure–Activity Relationship
QSPkR	Quantitative Structure–Pharmacokinetic Relationship
QSPR	Quantitative Structure–Property Relationships
QSTR	Quantitative Structure–Toxicity Relationship
SANN	Spiking Artificial Neural Network
SVM	Support Vector Machines
VLSI	Very Large Scale Integration
VS	Virtual Screening
